# CT of the chest can hinder the management of seminoma of the testis; it detects irrelevant abnormalities

**DOI:** 10.1038/sj.bjc.6603657

**Published:** 2007-03-20

**Authors:** G Horan, A Rafique, J Robson, A K Dixon, M V Williams

**Affiliations:** 1 Oncology Department, Box 193, Addenbrooke's Hospital, Hill's Road, Cambridge, CB2 2QQ, UK; 2 Radiology Department, Box 193, Addenbrooke's Hospital, Hill's Road, Cambridge, CB2 2QQ, UK

**Keywords:** testicular seminoma, staging investigations, CT chest

## Abstract

To evaluate the role of chest CT in the initial staging of testicular seminomatous germ cell tumours. All patients referred to Addenbrooke's Hospital with testicular seminoma from 1 January 2000 to 31 December 2005 were included and case notes retrospectively reviewed. One hundred and eighty-two patients with testicular seminoma were identified, with a median age of 37 years (range 19–74). Most patients had stage I disease (86%). Twenty-four patients had abnormal abdominal CT findings. One hundred and fifty-eight had normal abdominal CT findings but, on initial staging, chest CT reported abnormalities in 13 patients, which, on further follow-up CT were deemed to be irrelevant to the diagnosis of seminoma. There was a further patient with a normal CT abdomen in whom chest CT detected obvious metastatic disease, which was seen on chest *x*-ray. Overall 18 cases required additional investigations and follow-up for abnormalities subsequently found to be benign. There was a false-positive rate of 10% for initial staging with chest CT. This is the largest reported series of staging CT chest in testicular seminoma. In all patients with normal abdominal CT, normal chest *x*-ray and abnormal chest CT, subsequent follow-up investigations demonstrated that the lung lesions were incidental findings.

Testicular cancer is relatively uncommon accounting for less than 1% of all male cancers, but its incidence is increasing (Roth and Nichols, 1992). It affects mainly young men and carries an excellent prognosis when treated appropriately (International Germ Cell Collaborative Group, 1997). Initial staging is heavily dependent on radiological imaging and this plays a crucial role in subsequent treatment decisions (MacVicar, 1993). There is evidence that seminoma tends to metastasise in a serial fashion through the lymphatic system with contiguous disease spread from abdomen to mediastinum and then neck (Wood *et al*, 1996).

The relative use of computed tomography (CT) chest and chest *x*-rays and the optimum schedule for reassessment for stage I testicular tumours is controversial with practice varying considerably between centres (Nathan and Rustin, 2003). It has been our practice to obtain a CT chest in all patients as part of their initial staging work-up as recommended by Royal College of Radiology (COIN) guidelines (2000). CT chest is unquestionably more sensitive than plain chest *x*-ray at detecting small pulmonary metastases (Williams *et al*, 1987; White *et al*, 1999), but this has to be balanced against the risk of detecting benign abnormalities not seen on chest *x*-ray. In addition, there is the small risk of the increased radiation exposure.

This study evaluates the role of initial CT chest in the staging work-up of seminomatous germ cell tumours.

## PATIENTS AND METHODS

A retrospective analysis of 182 consecutive patients referred from the date of their orchidectomy from first January 2000 to thirty-first December 2005 with a histologically confirmed diagnosis of seminoma was performed. Data recorded from this 6-year-period included all initial staging investigations and subsequent radiological imaging, recording the clinical course and follow-up results.

All available imaging was reviewed again by a radiologist to ensure no thoracic lesions were missed at initial diagnosis. If a chest *x*-ray was also performed this was recorded as well. The Royal Marsden Hospital Staging Classification was used to stage the tumours (Horwich, 1991). The clinical notes were used to record clinical course and outcome. Our institutional practice was to discuss all new referrals at the Testis Multi-Disciplinary Team meeting (MDT) and the relevant pathology and radiology were reviewed before reaching a treatment decision. If an abnormality was found on chest CT and was felt to be of questionable significance, then a repeat chest CT was performed after a 6–12 week interval. The results would then be discussed again at the MDT and a decision made about further investigations or treatments.

Staging examinations of the chest, abdomen and pelvis were performed using CT at the local regional hospital or at the tertiary referral centre. Images were acquired following the administration of intravenous contrast medium, with the thoracic and abdominal images obtained at 25 and 70 s after the bolus injection, respectively. Two doses of oral contrast medium taken 1 h apart were also routinely administered. Follow-up examinations were performed at the tertiary referral centre following administration of oral contrast medium; the need to use intravenous contrast medium at this stage was left to the discretion of the reporting radiologist. Thoracic examinations were performed from lung apices to costophrenic recesses using contiguous 5–10 mm (or thinner) slices, depending upon the centre where the study was performed. Abdominal and pelvic examinations were performed using contiguous 10 mm (or thinner) slices from the dome of the diaphragm to the pubic symphysis. Examinations from regional hospitals were displayed as hard copy films with images of the thorax printed at mediastinal and lung window settings as this study preceded digital transmission from all hospitals. Examinations from the tertiary referral centre were available for review on a proprietary workstation.

## RESULTS

The median age was 37 years with a range of 19–74 years. The majority (86%) were stage I disease (Table 1). A further 4.5% were Stage IIA, 2% stage IIB, 6% stage IIC, and 1.5% stage III and above. Computed tomography chest was reported to reveal an abnormality in 12% (21) of the 182 cases. Chest *x*-ray was abnormal in four of these cases.

### Stage I disease – normal CT abdomen

**Figure 1:**
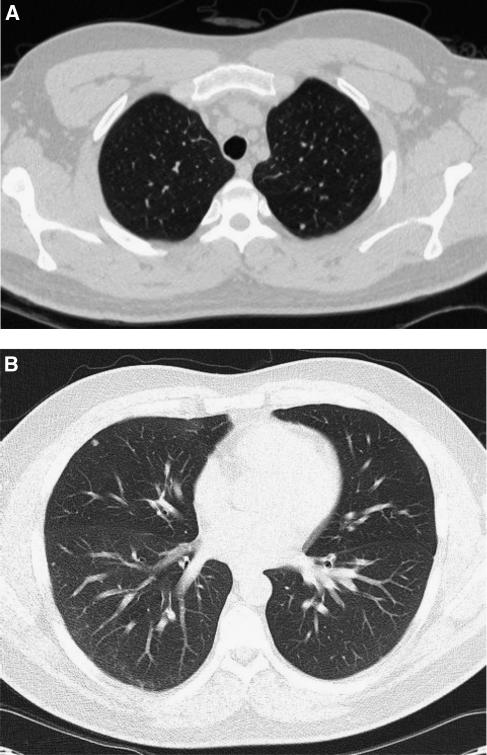
(**A**) lung nodule posteriorly left upper lobe; presumed granuloma, unchanged on sequential chest CT. (**B**) lung nodules anteriorly right middle lobe and laterally right lower lobe; presumed granuloma, unchanged on sequential chest CT.

**Figure 2:**
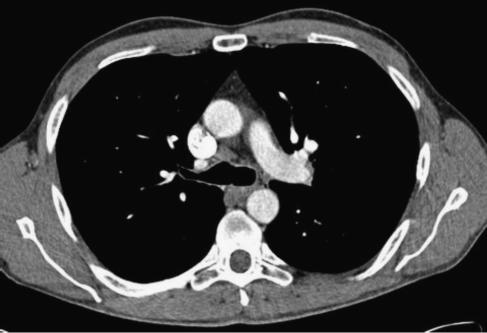
Precarinal lymph nodes 1cm in size, unchanged on sequential chest CT.

Of the 157 patients with Stage I disease, CT of the chest was reported as abnormal in 13 (8%), one of whom had an abnormal chest *x*-ray (Table 2). Of these 13 cases, nine showed parenchymal lung lesions of uncertain significance, typically lung nodules of <5 mm in size (Figure 1A and B). In two patients CT demonstrated enlarged intrathoracic lymph nodes of just over 1 cm (Figure 2). One patient had an abnormal chest *x*-ray and CT chest showed a 2 cm pleurally based lesion (unchanged after treatment, presumed granulomatous disease). And one case was found to have an anterior mediastinal mass that was surgically removed and confirmed to be a thymoma. Apart from this thymoma case, none of the follow-up CT chest examinations showed any change in the lung findings and these lung lesions were all deemed to be incidental. Thus in stage I disease, where abdominal CT and chest *x*-ray are normal, staging CT chest gives a false-positive rate of 100%.

### Stage II disease – abnormal CT abdomen

All 22 patients in the stage II group had abnormal abdominal CT findings. In five patients (23%) the chest CT findings were abnormal. In none of the five patients was the abnormality on chest CT found to be because of a malignant cause (Table 3). In the eight patients with stage IIA disease, two had abnormal chest CT findings. One showed a 2 cm superior mediastinal mass and the other case showed multiple pulmonary nodules all of <5 mm in size. These did not change after chemotherapy and were assumed to be due to granulomatous disease. In both patients the abdominal mass showed a complete radiological response. There were no patients with stage IIB disease with abnormal chest CT findings. There were three patients out of the 11 with stage IIC disease with abnormal CT chest. One had an abnormal chest *x*-ray which showed a right midzone subsegmental collapse. All three cases had abnormal CT chest, with parenchymal lung nodules of <5 mm in size. On follow-up CT these did not change, despite good responses of the abdominal disease to chemotherapy in all patients.

### Stage III and IV disease

**Figure 3:**
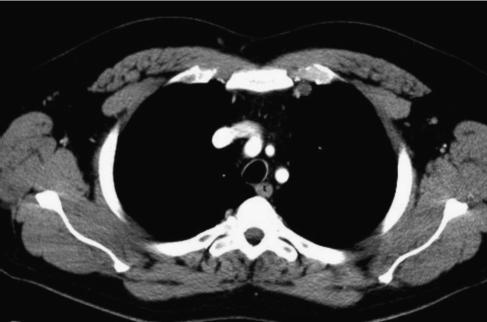
1.2 cm left internal mammary lymph node; unchanged on post chemotherapy chest CT, cause uncertain but it would be an unusual site for seminoma metastases.

Stage III disease, by definition, is associated with supradiaphragmatic lymphadenopathy. Two such patients were identified. One patient had bilateral 5 cm hilar lymphadenopathy associated with bulky para-aortic lymphadenopathy. These hilar nodes regressed on chemotherapy. The second patient had an abnormal chest *x*-ray with left basal atelectasis. Computed tomography chest showed a large anterior mediastinal nodal mass and a left internal mammary lymph node (Figure 3). The CT abdomen was normal. The mediastinal nodal mass regressed completely with chemotherapy. There was one stage IV case with a normal chest *x*-ray. Computed tomography of the abdomen revealed a 6 × 7 cm aorto-caval nodal mass, enlarged common iliac lymph nodes and on chest CT, small nodules over both hemidiaphragms and in the right middle lobe.

The false-positive rate for CT chest in the initial staging of seminomatous germ cell tumours of the testis is 10%. The false-negative rate is 0%. The positive predictive value of CT chest is 0.14, and the negative predictive value is 1. The sensitivity is 1 and the specificity is 0.89. Overall lung metastases were detected in 1.6% of cases.

## DISCUSSION

Seminomatous germ cell tumours are primarily a disease of young men and with appropriate treatment they have an excellent prognosis with a cure rate of 95%. These patients are likely to live for decades after treatment and the risk of radiation-induced second solid malignancies that manifest 12–25 years post-exposure is a real threat. This has largely been related to therapeutic irradiation (Travis *et al*, 1997; Zagars *et al*, 2004). Under the ionising radiation (medical exposure) regulations (2001), there is a legal requirement to keep radiation dose as low as reasonably achievable, and the radiation exposure from a CT chest is approximately equivalent to 400 chest *x*-rays, equivalent to a risk of radiation-induced second malignancies of 1 in 2000 (RCR, 2003). If chemotherapy is increasingly used (Oliver *et al*, 2005); diagnostic imaging will assume greater importance. Chest CT has been established as the imaging modality of choice for staging patients with teratoma of the testis (Bradey *et al*, 1987). Once the histological diagnosis of seminoma is confirmed, formal radiological staging is required.

There is good evidence for contiguous lymphatic spread in seminoma (Wood *et al*, 1996). In a series of 31 patients with supradiaphragmatic metastases of which 11 were seminoma, they confirmed the contiguous nature of disease spread from abdomen to chest and neck in seminoma. Supradiaphragmatic disease tends to be contiguous spread from the retroperitoneum through the retrocrural groups and into the posterior mediastinum where the paraoesaphageal and subcarinal lymph nodes are most frequently involved. Pulmonary metastases without abdominopelvic lymphadenopathy are rare. This would support the theory that seminoma tends to metastasise in a relatively predictable fashion through the lymphatic system and a normal CT abdomen and pelvis could exclude the need for a CT chest, if chest *x*-ray is normal.

A number of studies have been published that address the issue of thoracic imaging in non-seminomatous germ cell tumours (NSGCT) specifically. In one series (Harvey *et al*, 2002) with 168 patients on surveillance, eight developed thoracic metastases, all of which were visible on chest *x*-ray. Another study (Fernandez *et al*, 1994) highlighted the difference in sensitivity between chest *x*-ray and CT, comparing CT chest at initial diagnosis to chest *x*-ray in 117 patients with NSGCT. Twenty-one patients had thoracic metastases on CT, of which five were missed on plain chest *x*-ray. This could have potentially altered treatment in two of these five patients. The role of CT and chest *x*-ray in 207 germ cell tumour patients has been evaluated (White *et al*, 1999) and found intrathoracic metastases in only 1% of seminoma patients. However, they still advised CT chest in the initial staging. The North American literature advocates chest *x*-ray alone for both staging and follow-up surveillance of seminoma where a CT abdomen is negative (Steinfield and Macher, 1990; Moul, 1995; See and Hoxie, 1993).

Computed tomography abdomen is used in the initial staging of seminomatous germ cell tumours of the testis and usually CT chest is performed at the same time. Computed tomography is now often done before histology results are available so it is not practical to omit staging chest CT. There has also been a major evolution in chest CT, now that many centres have modern multidetector CT. Using high resolution (HRCT) algorithms, CT chest can be reconstructed for optimal demonstration of lung parenchyma. The number of thin sections has increased substantially and the number of small incidental findings has correspondingly increased (Hayward, 2003). Some of these incidental findings represent pre-existing sarcoid, which is a well-recognised trap when staging young people (Remy-Jardin *et al*, 1991).

We question whether the CT chest should influence management and delay treatment if the abdominal CT is normal, as there is a significant false-positive rate of 10% for chest CT in our single institution series. The issue of false-positive CT chest is a serious one, as the delay between the initial CT and the subsequent follow-up CT in 6–12 weeks can be psychologically distressing for both patients and their families. For those cases that could be stage I, treatment cannot start until the lung abnormalities have been clearly defined as incidental or malignant, as the treatment regimens are so different. We found that for stage I disease, 100% of chest CT abnormalities were determined to be incidental on subsequent follow-up. There was one case with an anterior mediastinal mass which was correctly identified as a thymic mass and surgically removed. No abnormality was related to the diagnosis of seminoma.

We conclude that for patients with normal abdominal CT findings and thereby presumed low stage tumours, any abnormality in the chest CT should not necessarily influence management or delay treatment. Computed tomography chest has unacceptable false-positive rates and should not precipitate further additional invasive investigations. This would avoid delay in diagnosis and treatment caused by the unnecessary investigation of incidental lung nodules.

## Figures and Tables

**Table 1:** Patient demographics

**Stage**	**No. of patients**	**% cases**
I	157	86
IIA	8	4.5
IIB	3	2
IIC	11	6
IIIO	1	0.5
IIIB	1	0.5
IVL	1	0.5

Age: median, 37 years; range, 19–74 years.

**Table 2:** Analysis by stage of intrathoracic disease from CT thorax results

**Stage**	**Abn CT Abdo**	**Abn CT Chest (no. cases)**	**Type of lung lesion^a^**	**% Incidental on F/U CT**
I	0	13	Type 1=69%	
			Type 2=15.5%	
			Type 3=15.5%	100%
				
IIA	2	2	Type 2=50%	
			Type 1=50%	100%
IIC	3	3	Type 1=100%	100%
IIIB	1	1	Type 2=100%	Not applicable
IIIO	0	1	Type 2=100%	Not applicable
IVL3	1	1	Type 1=100%	Not applicable

a Types of lung lesions are: 1**=**parenchymal lung mets, 2=intrathoracic nodal mets, 3=other lesions

F/U=follow-up.

**Table 3:**
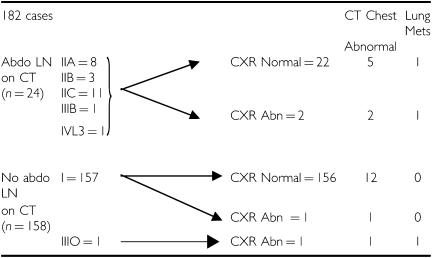
Analysis from CT abdomen results
